# Medical and Philosophical Intelligence

**Published:** 1818-04

**Authors:** 


### MEDICAL AND PHILOSOPHICAL INTELLIGENCE.

ON Monday, the 9th of March, the *Medical Society of London* held its anniversary meeting, when the following officers and members of council were declared elected for the ensuing year :—

*President*—Dr. Walshman.

*Vice-Presidents*—Dr. Merriman, Dr. Haighton, Mr. George Young, Mr. Whateley.

*Treasurer*—John Andree, esq.

*Librarian*—Dr. Hancock.

*Secretaries*—Dr. Uwins, Mr. Blegborough.

*Registrar*—Mr. Pettigrew.

*Council*—Dr. Adams, Dr. Clutterbuck, Dr. Blegborough, Dr. Ley, Dr. Hooper, Dr. Fryer; Messrs. Pettigrew, Vance, Leese, R. Adams, Saner, Johnston, Sutliffe, Stevenson, Hooper, Seaton, S. Griffith, Field, Dunlop, W. Griffith, Dugdale, Robinson, Radford, Hilliard, Brown, T. Bartlett, Brougham, Newby, Winter, Wadd.

*To deliver the Anniversary Oration*, 1819—Mr. Whateley.

After which, the oration was delivered by Dr. Uwins. This consisted of a general statement of the orator's sentiments on the present condition of medical theory and practice. Dr. U. commenced his discourse by apologizing for the expression of any difference of opinion that might exist between himself and several members of the Society. He then proceeded briefly to reply to the arguments which have been urged against the assumed progress of medical science; dwelling particularly upon the increased demands which the present times of luxury make upon medical skill, and remarking upon the comparatively little faith reposed now in the virtues of medicine: both circumstances, he urged, necessary to be taken into account, before any comparison could, with fairness, be instituted between the ancient and modern state of the healing art. After these preliminary remarks, the orator entered upon the more immediate business of his discourse, and made a general division of the leading doctrines and practice of medicine into three parts. He first alluded, with some severity, to those views of the science which attribute every thing of a morbid nature to the agency of the liver. He then proceeded to descant upon the extravagancies of the "digestive-organs" doctrines; and, lastly, stated it to be his opinion, that, in combating the stimulating and antispasmodic principles of the Brunonian and Cullenian schools, some pathologists of the present day had carried their notions too far on the subject of vascular plenitude, and congestive conditions, as explicatory of morbid phenomena.

In the recapitulation of his discourse, Dr. Uwins protested against the inference which might be drawn from his remarks, that he meant any thing further than to object to the abuse of the several doctrines upon which he had animadverted; and observed, in conclusion, that, moderately and restrictively applied, there is much of what is true and useful in all of them. The errors of theorists, he argued, even in the present improved condition of medical enquiries, principally consist in aiming at too much, and endeavouring to connect, by one bond of union, what, in its very nature, must necessarily be constituted of separate and detached parts.

The auditors, we were happy to observe, were very numerous, and the visitors highly respectable. Among the latter, we saw some physicians, not members of the Society; and one, at least, of the fellows of the College. The Society adjourned, after the oration, to the Globe Tavern, Fleet-street; where a large party dined, and spent the evening with much convivial harmony.

On Monday, a very interesting paper from Mr. White, at Bath, was read. The subject was on the Stricture or Contraction of the Rectum, unconnected with Scirrhus. The practical remarks are so important, that we trust the paper will be printed in the next volume of the Transactions of that Society.

---

*To the Editors of the London Medical and Physical Journal.*

Gentlemen,—I request you will give me an early opportunity of correcting an error inadvertently made in my paper on Arsenical Tests, which had escaped my observation, until Mr. Kerr, in the last Number of your Journal, did me the favour of pointing it out. The mistake was occasioned by the misplacing the words *former* and *latter*: if these be transposed, the meaning I intended will be conveyed. The sentence will then run thus—"The latter becoming gradually of a brown colour, while the former assumes a blackish hue." I take this opportunity of stating that, at the time I sent my last communication, I was not aware the black appearance of the phosphate of silver had been remarked. I think it right to mention this circumstance, as there is *apparently*, in my paper, a want of acknowledgment of the experiment made by Dr. Ayrton Paris. Upon the subject of the ammoniaco-nitrate of Mr. Hume occasioning no precipitate with the phosphate of soda, I confess myself ignorant; at the same time, I believe I may add, it was a circumstance not generally known; and in this opinion I am confirmed by its not having been noticed by Dr. Thompson, in his Sketch of the latest Improvements in the Physical Sciences: from whose scientific Journal I extract the following—"The employment of nitrate of silver as a test for arsenic, does not seem to be known in Germany. This test, first pointed out by Mr. Hume, but much simplified and improved by Dr. Marcet, is certainly very delicate; and, when the precautions suggested by Dr. Marcet are attended to, does not seem liable to ambiguity."*

I remain your obedient servant,

*Halesworth; March* 12, 1818. W. J. Crowfoot.

---

* Vide Dr. Thompson's Annals of Philosophy, Jan. 1818.

We are favoured with the following communication from the medical officers of the Small-Pox Hospital, who express a wish to give it every possible publicity.

*Fenstanton, St. Ives, Hunts; Dec.* 12, 1817.

Sir,—At a vestry meeting held a short time ago, in this parish, for the purpose of considering the propriety of inoculating the parish, the small-pox having made its appearance in the neighbourhood, it was stated, in opposition to vaccination, that that system of inoculation had lately come into disrepute, and, especially, that it had been *discontinued* by the Small-Pox Hospital. Being extremely doubtful of the truth of this statement, and anxious to promote what I had been led to think a great benefit to the human race, I requested my brother, Mr. F. Bourdillon, 14, Russell-street, Covent-garden, to call at the Small-Pox Hospital, and ascertain the fact. On his doing so, he informed me that the case was very different from what had been stated at our meeting; that, instead of vaccination having been discountenanced by the Hospital, they had vaccinated eight hundred more persons this last year than in any former one, and had given public notice that they did not intend to inoculate for the small-pox any more of their out-door patients. This information has already had a good effect in our parish; but I take the liberty of writing to you, Sir, that I may be the means of doing further good, by having the statement under your hand, and giving it a wider circulation; as, I am sorry to say, a considerable degree of prejudice seems still to exist, in this neighbourhood, against vaccination. If, therefore, you would be good enough to favour me with an answer to this letter, stating the above facts, and any thing else which you may think proper, it will be both a benefit to the community, and an obligation conferred on, Sir, your obedient servant,

Thos. Bourdillon,
*Vicar of Fenstanton.*

P. S.—Perhaps you would not object to my inserting an extract from your letter in our provincial paper, the Cambridge Chronicle, if I should judge it expedient. May I take the liberty of adding, that, the sooner I am favoured with your answer, the better it may, perhaps, be for the interests of this parish.

*Answer.*

*Hatton-garden; Dec.* 13, 1817.

Rev. Sir,—I cannot lose a post in acquainting you, that the report given by your relation, Mr. B. was, in all respects, correct. Nothing can be more proper than the proposal you make to advertise in the neighbouring newspapers. Joseph Adams.

---

*To the Editors of the London Medical and Physical Journal.*

Gentlemen,—In three or four days after a suspicious intercourse, in the summer of 1815, I perceived a small superficial circular sore upon the inner surface of the prepuce, which I supposed to be a chancre, and touched it twice a day with lunar caustic. It healed in the course of a few days; during which time I took a little mercury, but left it off as soon as the sore healed. I had not been rid of this sore a month, before a second appeared, (without any farther connexion,) about the third part of an inch from the first, which readily got well, with the daily application of dry lint only. From this period till August 1817, I was from time to time visited by these sores, which gave me no trouble, excepting that of applying lint to them twice a day.

Thinking that, perhaps, a course of mercury would rid me of these unwelcome visitors, I took about 6 grs. of blue-pill every day for a fortnight; when one of the glands in my right groin began to enlarge, which frightened me, and caused me to consult a surgeon. He was of opinion, after having heard my story, that my disease was not venereal, and advised me to take no more mercury, but to use cold lotions, leeches, &c. all of which were of no use: the gland continued to enlarge. On my next visit to my surgeon, he changed his mind, and advised me again to have recourse to mercury. In consequence of this, I rubbed in ℥j. of ointment, and took ʒss. of blue-pill, alternately, every day for a month, without its having the least effect upon my mouth, though my constitution was considerably affected by it, and I was troubled with constant head-ache. In October the gland suppurated, was opened, and readily healed. I continued taking the blue-pill, about three times a week, for some time.

March, 1818.—The gland is still large and hard, but gives me no pain. There is a hard, deep-seated tumour, about the size of a hen's egg, on the same side, about an inch from the symphysis pubis, which hurts me much when pressed upon; and a gland in my left groin has enlarged to the size of a pigeon's egg, is sore, and has been stationary for several weeks.

E. O. N.

⁂ We are perpetually receiving letters of the above description, and, where cases are complicated, we never fail to insert them. They make a useful question to exercise the thoughts of practical men, and often prove serviceable to the individuals, and consequently to mankind at large. The present is admitted on account of its brevity, and to give us an opportunity of requesting that such querists would consult the best practitioners before they propose their questions for the public. The ulcer which appeared a month after the first could not have been venereal; nor is there the shadow of a reason for supposing any of the succeeding ones were of that description. The date of the swelling in the glands not being mentioned, we shall give no opinion of its probable origin, but advise the writer (who, we suppose, by his Arabic characters and anatomical language, to be medical,) to consult Mr. Hunter's comprehensive work, in which he will find an answer to all his questions.

*To the Editors of the London Medical and Physical Journal.*

Gentlemen,—Some time ago, an extract from a letter of mine appeared in your Journal, speaking of the anti-syphilitic properties of nitric acid. Since that, I addressed you in reply to Dr. Scott's paper on the nitro-muriatic acid: in this letter I related the observations I had made on this subject, and endeavoured to shew that the doctrine I had held in the paper from which the extract was taken agreed with that of Dr. S. where he speaks of the copper-like taste experienced from nitric acid, after having taken mercury. This paper, Gentlemen, has not yet appeared in your valuable pages. It might not, perhaps, have come to hand; or, if it did, probably was not thought worthy: if the former be the reason, I am sorry for its loss; if the latter, I am perfectly satisfied in having discharged my duty to the profession, by making such enquiry into a subject of the first importance as my abilities would allow. Whatever imperfections may appear in it, you will consider it was not written to undergo the severity of criticism: but I trust in your politeness to know what is the destination of the fruit of my labours. Your obedient, humble servant,

H. Ward.

*** We are much obliged to Mr. Ward for his former communication, and trust, by inserting the present, we shall at once show the coincidence of his remarks, and make our own apology.

---

*Extract of a Letter, dated Bristol.*

*To the Editors of the London Medical and Physical Journal.*

Gentlemen,—In your Number for this month (February), Dr. Uwins, in a paper on the "Connexion of Tabes Mesenterica and Hydrocephalus," says—"Now, in mesenteric atrophy, there is nothing present to authorise the predication of any primary disease in the first passages. The disorder does not consist in faulty digestion, but in defective assimilation: there is a clean tongue, there is a good appetite, there is a due secretion of the gastric juice; but the *glandular* condition of the frame perverts the chyliferous process, or at least prevents the possibility of the chyle finding its way duly into the blood-vessels. Hence the disease, with all its consequences, either necessary or immediate, or remote or contingent; and hence the *modus operandi of its termination in hydrocephalus,* when that, as it not seldom does, happens to be the consequence of the original malady. It is, in this case, the lymphatic system which seems to take on the sympathetic irritation; and hydrocephalic effusions are the consequence, not of digestive derangements, but of glandular torpor."

Now, admitting hydrocephalic effusion to be the last link in a chain of morbid condition of parts, and the glandular condition of the mesentery to be *the original malady*, on which the chain is suspended,—what are those intermediate links of morbid condition, which are concealed from our view under the expression—"*modus operandi of its termination in hydrocephalus?*" Are they designated by—"It is, in this case, the lymphatic system which seems to take on the sympathetic irritation; and hydrocephalic effusions are the consequence, not of digestive derangements, but of glandular torpor?" Then I am to understand, that glandular torpor is one link, and sympathetic irritation of the lymphatic system another link, in the chain of causation. My powers of ratiocination are too feeble to see the dependene of these phenomena, admitting their existence.

*February* 11, 1818. ———.

---

*Explosion in a Coal-mine in the County of Durham.*—The following account of another of these fatal accidents is taken from the Tyne Mercury, December 23:—"On Thursday, Dec. 18, an explosion of fire-damp occurred in the Plain-pit at Rainton-colliery, near to Houghton-le-Spring. The total number of lives lost amounts to twenty-six,—ten men and sixteen boys. The explosion took place at three o'clock in the morning, before the hewers had descended the pit; and, from this circumstance about one-hundred and sixty lives have been preserved. Every exertion was made to render assistance to those in the mine, and we regret to add, that two of the above men fell a sacrifice to their humane endeavours, having been suffocated by the impure air. The viewers and agents were extremely active, and had nearly shared the same fate. A correspondent who visited the pit says, 'After particular enquiries, I found that though Dr. Clanny's safety-lamps have been generally employed in the collieries of Lady F. Ann Vane Tempest, it so happened that this pit has heretofore been so free from fire-damp, that no safety-lamp had ever been used in it.' All the dead bodies were got out by Sunday; thirteen were buried at Houghton, and four at Chester, on Saturday evening; and the remaining nine were interred at the former place on Sunday."—*Annals of Philosophy.*

---

An old Pauline, at present engaged in the government of that venerable school, by his situation in one of the livery companies, has favoured us with a copy of the regulations published early in the sixteenth century. Among the rest, we meet with one law, which induced him to present us with the whole, in consequence of our remarks on the leprosy and venereal disease, as confounded with each other, and with many other complaints.

"If the master should be too old for his office, or be stricken with the lepry or French pox, he is to retire with a pension."—By this, it is evident, that no disgrace was attached to the disease at that time; because, under all such circumstances, a smaller allowance was made him than that of the pension on retirement.

---

The Abbé RAYNAL remarks, that the English are so truly commercial as to mix commerce with their philosophy, and even with their religion. As a proof of this, he instances Mr. Locke, who, among his arguments in favour of converting the Indians, adds, that, by being thus induced to cover their naked bodies, they would add to the consumption of British manufactures. At present, a new question arises:—How far the encouragement to our cotton manufactures has proved injurious to the health of the children engaged in it? This is an argument much insisted on by Mr. Maitland, chairman to the Wool-committee, in the House of Commons. In a well-written pamphlet, he dwells much on this, and on the general healthiness of those engaged in the manufactory of wool. We shall be thankful for any information on this subject, from our correspondents settled among those engaged in either of these occupations.

---

*A Plan for observing the Meteors called Fire-balls;* by NEVIL MASKELYNE, D.D. F.R.S. and Astronomer Royal.—Five meteors, of the kind which, from their appearance, are generally called fire-balls, have been seen of late, in the space of a few weeks, viz.—on August 18, Sept. 26, October 4th, 19th, and 29th; which seems to indicate that they appear more frequently than is commonly imagined. The curious and extraordinary appearances which they exhibited, show them to be deserving more attention than has been hitherto given them. For want of a series of proper observations, little progress has been made towards accounting for their phenomena. The greater part of those who have seen them, not being previously acquainted with the circumstances they ought to attend to, have made observations too imperfect to answer that purpose. It is, therefore, to be wished that all persons, who may happen to see a meteor, would attend to the following particulars, and set down their remarks as soon as they can after they see it, while the impression made by the meteor is full and fresh in their memory, before it is vitiated by their own after-thoughts, or the accounts received from other observers. Such after-thoughts may be of great use; but their own genuine original observations are chiefly to be wished for by any one who is to calculate the tract of the meteor.

The particulars to be attended to are these—

1st. The precise time of its appearance.

2d. Its apparent altitudes and bearings at its first appearance, at its greatest elevation, at its bursting, and at its disappearance.

3d. Its figure, and the diameter of the body when at the greatest apparent altitude, compared with that of the sun or moon at the same altitude; the brightness and colours of its light, and the degree of illumination which it gave; and to make a sketch or drawing of the appearances before and after it burst, or any other of its appearances.

4th. Whether both the body and the tail burst; and how many parts this bursting produced; and whether this happened before or after it arrived at its greatest apparent altitude; the length of the tail before the meteor burst; and, indeed, every alteration of its length they observe; whether the meteor appeared very faint at first, and gradually grew brighter, or appeared very bright at once; and whether it was extinguished suddenly, or by degrees.

5th. How long the appearance lasted.

6th. Whether a sound or sounds (as of an explosion) was heard some minutes after its disappearance, and how long, and from what point of the compass they thought it came.

7th. The bearing and distance of the place of observation from the nearest market-town should be put down.

N.B. As sound moves only at the rate of thirteen miles in a minute, the observer should patiently wait for at least eight or ten minutes, listening for the sound; for all meteors appear to be very many miles indeed nearer to the observer than they really are.

*Remarks.*—Curious persons may avail themselves of observations made even by the most illiterate, by causing them to trace, with a stick, the path which the meteor described in the heavens, according to the best of their recollection. The observations would be better made, if you accompany the person to the very spot where he saw the meteor; for there the neighbouring objects,—such as roads, houses, or trees,—will much assist his memory.

The apparent altitudes of the meteor are best found by a quadrant (a common wooden one of three inches radius, divided into degrees, will suffice), which the person should direct to the points in the heavens where the meteor appeared to him, if he saw it, or even to such points where the illiterate person, above mentioned, pointed. In like manner, its bearings should be found by a compass.

To ascertain how long the appearance lasted, he should trace over its path in the heavens, with its proper velocity, while another person observes the time by a watch or clock that shows seconds; or by the number of swings of a temporary pendulum, made by a musket-ball, or any small weight, suspended by a string of thirty-nine inches long from the centre of the ball or weight, which will swing seconds. Without some such method as this, they will be apt to estimate the time much longer than it is.

It would be well if those persons who happen to see a meteor would put down the time, by their watch, when it first appeared, or was at its greatest altitude, or burst, or disappeared, and again when they hear the sound; and, as common watches are liable to vary much in a few hours, that they would, as soon after as may be, find the error of their watch by comparing it with a good regulator; for, if the exact times could be had at different places, the absolute velocity of the meteor, the velocity of the sound propagated to us from the higher regions of the atmosphere, and the longitudes of places might be determined.

Even in cloudy weather, it might be useful to note the times of accidental explosions, or any unusual sounds heard, with the points of the compass from which they are thought to come, whether in the day or night, and of sudden illuminations of the sky in the night; as they may prove afterwards to have been owing to meteors, and will serve some of the purposes above mentioned.

These meteors generally leave a visible tract of faint light behind them, which gives time to observers to ascertain the path, either by the stars near it, or the observations of altitudes and bearings. Meteors are sometimes seen in the day light.

It may not be amiss to apprise observers, that estimations of altitudes made without an instrument are very uncertain, owing to the apparent figure of the sky being the segment of a sphere, whose centre is greatly below the surface of the earth; so that persons will be apt to judge an object, which is near the horizon, to be much higher than it is: at 23° of altitude, they might think it at 45°; and to be in or near the zenith, when, with an instrument, it would be found 10 or 20 degrees from it. This points out the necessity for observers to mention, whether they estimated their altitudes, or observed them with an instrument.

---

We copy the following from the *Journal de Médécine*, to show how imperfectly our continental neighbours are acquainted with experiments made in the British metropolis.

A woman, at *Leneux*, *Departément de l'Ourthe*, in France, aged forty, died after a short illness, which was manifested by a considerable tumefaction of the genital parts, by uterine hæmorrhages, vomiting, and abundant purgation. This woman confided to two of her neighbours, that her illness was occasioned by powdered arsenic, which her husband, at the moment of enjoying the conjugal rights, had himself insinuated into the parts.

This was made known to the mayor, who ordered the body to be opened by the proper officers. They declared that they found the vulva and vagina in a state of gangrene, the abdomen much distended with air, and the intestines inflamed and gangrenous. The culprit was arrested, convicted, and executed.

In the Acts of the Society of Medicine of Copenhagen, a similar crime is recorded, committed also by a peasant. In this last case, although some small pieces of arsenic were found within the vagina, yet some, doubting the possibility of this species of poisoning, the magistrates consulted the Copenhagen College of Medicine, which, to decide the question, made the following experiments:—

The 16th of April, at six P. M. a bolus, prepared with honey and half an ounce of arsenic, was introduced deeply into the vaginæ of two mares. In half an hour, they gave evident signs of pain, often urined, and alternately rose and laid down. At ten, there was swelling and redness of the parts. The next morning, they refused to continue standing: the tumefaction and redness of the vagina and vulva were considerable; the evacuation of urine was less frequent; but the discharge of fecal matter was natural. They had no fever; but appeared sad and dejected.

One of the mares was abandoned to the action of the poison; to the other was administered means of relief, which consisted of emollient and lightly sedative injections, which calmed all the symptoms, and restored the animal to health.

In the mare for which no relief was applied, the inflammation and tumefaction of the vulva became extreme, and the part was covered with phlyctænæ.

The fourth day, the pulse beat no more than thirty in a minute, and death occurred about noon.

On opening the body, the neck of the uterus was found swelled and gangrenous, and it contained coagulated blood. There was, besides, an effusion of bloody serum in the abdomen, and traces of inflammation in the stomach, intestines, lungs, aorta, thoracic duct, &c. The pericardium contained much bloody serum.

Now it has long since been confirmed, that arsenic, and many other poisons, applied to any parts not protected by the common cuticle, is more certainly deleterious than when taken into the stomach, and produces symptoms very similar in that organ; but, in the latter, it is sometimes vomited up, after being enveloped in mucus, by which the stomach is molested.

---

The following papers, from the *Gazette de Santé*, show how little the French have improved by the remarks of our pathologists on Hydatids, and on their conversion into calculous matter.

*Observations on a Wound of the Testicle, accompanied with the Formation of a Bony Concretion.* By —— Manoury, M.D. at Vernon.

N——, of the parish of Chambrai, about ten years ago, bruised his testicle: this was followed by a schirrous swelling, of which the patient took no notice, as he suffered very little from it; but, during the year 1812, he hurt himself again with his spade, the handle of which hit the same testicle very hard. Pain, inflammation, and swelling, now came on; a considerable suppuration was formed, and opened of itself; the size of the testicle diminished, but still remained harder and larger than the other; and, when I saw him, there was a fistulous sore, with adhesion of the skin of the scrotum to the edge of it. The man being in good health, and feeling no pain, I thought it best to leave him to nature, and apply nothing but emollients.

After having remained some time in the hospital at Vernon, our patient, finding himself strong enough, returned to his usual occupations; but he soon came to me again, telling me that he felt a *bone* on the edge of the sore. I laughed at him, and told him it was impossible; but that, to satisfy him, I would look at it. It was with the greatest surprise that I perceived and touched a white hard substance, which I extracted with difficulty, and which has really the appearance of a bone. On the following days, I extracted, with more or less difficulty, similar portions, which were fixed in the substance of the testicle, and which I send to the Society.

It is for the chemists to analyse this substance, which I do not conceive to be a real bone, but a calcareous phosphate, which, naturally existing in the seminal fluid, has thickened (I know not how), and, by length of time, has assumed a bony appearance.*

This extraordinary, and perhaps singular, state of the testicles, did not prevent this man from getting two children since the beginning of his complaint; but this may be easily accounted for, as the other testicle is quite sound.

Messieurs Lelarge,—one physician at Pontoise, and the other mayor of Chaumont, near Gisors,—saw and touched one of these white bodies, still fixed in the testicle, and expressed the greatest astonishment.

I have not seen the patient for a very long time, though he promised to come back.

*Reflections by the Editor of the Gazette de Santé.*

This accidental formation of portions of bone is certainly not uninteresting, but it is not so uncommon as M. Manoury seems to think. The bony tissue developes itself accidentally in all the other tissues, and particularly in the serous membranes: examples are so common that it is almost useless to quote them. It was probably in the tissue of this kind, which, under the name of *tunica vaginalis*, forms a cover for the testicle, that the portions of bone, which M. Manoury extracted, were formed. As to the cause of this formation, it is concealed with that of the other phenomena of the formation and growth of our bodies, in a mystery which the illustrious Bichat did not explain, but of which he made known a general law, in treating of *organic sensibility*. The explanation of M. Manoury is perfectly inadmissible, as it is in contradiction with the laws of life. The elementary parts which compose the seminal fluid could not have been separated, but by its decomposition; from which would have resulted a putrefaction, which, far from favouring the re-union of phosphate of lime, would have produced very serious symptoms, and quite different from those observed: but it is needless to dwell on an explanation to which the author seems to attach no importance. An example of an osseous production in the tunica vaginalis is to be found in the Treatise of the Diseases of the Urinary Passages, by Chopart, part ii. p. 69; and Mr. Baillie saw a case, in which bony fragments were floating, disengaged, in the cavity of the tunica vaginalis. No fact of this kind, however, is so remarkable as that which is related in the New Observations on the Practice of Midwifery, by Peter Amand, 8vo. Paris, 1714, p. 80; for, in this case, the bony tumour was as large as the head of a child of six months! Nevertheless, on extirpating it, the testicle, as almost always happens, was found perfectly sound; which would explain how the man, of whom M. Manoury speaks, might have got two children, even though he had not had a second testicle.

---

* An examination of this substance has confirmed the opinion of M. Manoury.

We have to lament the loss of that venerable patriarch in medicine, the late Dr. COGAN. For the following memoirs, we are principally indebted to the *Monthly Magazine*. This learned physician was descended from a respectable family of dissenters, who had been settled for a considerable time in Northamptonshire. He was born in that county in the year 1736; and his father was a respectable apothecary in one of the many towns with which this extensive district is so profusely studded. He had the good fortune to be sent to Kibworth, in Leicestershire, under the tuition of Mr. Aikin. That he improved greatly beneath the eye of this worthy and respectable man, there can be but little doubt; indeed, he was ever duly impressed with a high sense of his merits, his services, and his attention; and he was always accustomed, throughout the whole course of his life, to lament that he was not suffered to remain a few years longer under his roof.

On his return home, as he proved a prudent and considerate young man, it was determined by his father that he should become a minister of that respectable sect, in the principles of which he had been brought up from his infancy: Mr. Thomas Cogan accordingly embraced this mode of life, as a profession in which he could not only obtain a respectable livelihood for himself, but, at the same time, might prove highly serviceable, by his labours, to the instruction and edification of those of his own persuasion. He accordingly engaged in the usual initiatory studies; and at length became what the Scotch presbyterians term a *probationer*,—that is, after being duly approved of, he was to preach the Gospel in any meeting to which he might receive a call by the voice of the congregation.

Those who knew Dr. Cogan, will not wonder that he never became popular as a preacher. To use a common expression, he was made of different stuff. Profound learning, close reasoning, and well-connected periods, without unnecessary ornament, are better suited to the closet than the pulpit; and, that such were Dr. Cogan's endowments, his late publications incontestibly prove. Having been engaged as a subsidiary at the English church at Amsterdam, the current of his thoughts were naturally directed to medicine by his neighbourhood to the then-celebrated University of Leyden. Nor is it improbable, that the recollection of the illustrious Boerhaave's original destination to the church, and his subsequent success in medicine, might have influenced a young adventurer.

After a residence in Leyden, he pronounced and published his inaugural dissertation on Vitality, a subject he always kept in view, and turned so much to the advantage of mankind, and the honour of his native country.

Having now taken his degree, he married Miss Green, the daughter of a respectable merchant at Amsterdam, who had long been the object of his choice; and with this lady he obtained a very considerable fortune: she also appears to have been a very amiable and a very accomplished woman. He resided, during his long stay in Holland, in various cities of that republic (as it was then called),—particularly Amsterdam, Leyden, and Rotterdam. He was accustomed to practise generally as a physician; but seems to have devoted himself chiefly to the obstetric art; and was frequently called in to assist in the families of wealthy and distinguished personages.

He appears at one period to have resided, for a considerable time, at Zuylestein, in the parish of Leersum, not far from Wyk, a place connected with English history, and still appertaining to an English nobleman.* This was at a time, we believe, when he had abandoned all his professional avocations, and was enabled to live like a private gentleman. He himself, at a latter epoch of his life, describes his place of abode, in a letter to a friend,—of which the following is an extract:—

"This mansion was, in former days, the occasional residence of William the Third; and, for about three years, the constant habitation of your humble servant. It is one of the four hunting seats belonging to that prince,—which are situated in these quarters, at such convenient distances from each other, that, let the hare and the hounds, the fox, the partridge, the boar, and the wolf,—that, in extreme winters, visit these regions,—lead him where they please, they could not lead him far from home. The others are the palaces of Loo and Soesdyke, also in the province of Utrecht; and Dieren, in the province of Guilderland.

"Zuylestein is now the property of the Earl of Rochford, who is descended from the Nassau family. The late earl, during a visit he paid some years ago, was so charmed with the rural situation of this palace, that he proposed to himself the pleasure of paying it frequent visits. The design was not put in execution, and the reputed salubrity of the soil was, at that period, a powerful inducement to make it my abode.

"As I (afterwards) passed by its venerable turret, rising above the lofty trees, that had so frequently and affectionately beckoned me home from every rural excursion, I felt an agreeable—a disagreeable—compound sensation, at being at once an intimate acquaintance and an inadmissible stranger.

"It is the general opinion that kings never sleep! It is reported, that they lie on beds of thorns, and that their brows are encircled with pungent cares which keep them constantly awake. If this be the case, they must bring their thorns and their nightcaps with them, for I never slept more comfortably in my life than in the very bed where it is supposed his majesty* tossed and tumbled about, like (with all due respect to majesty) a porpoise portending a storm. The only thing that kept me awake the first night, was the splendour of the furniture. I thought it was a pity to close my eyes upon rich tapestry, silk damask curtains, chairs and settees of crimson velvet, fringed with gold. Morpheus,

* The Earl of Rochford. † William III.

however, finally subdued Plutus; and familiarity, with all this grandeur, bred neglect, but not contempt."

Nothing can be more characteristic of the simplicity, archness, and good temper, which formed the Doctor's character, than the above extract.

About the year 1772, Dr. Cogan, accompanied by his wife, returned to London, and commenced the obstetric practice, under the auspices of Dr. Foord, of the city. Though he met with every encouragement, yet a residence in the Old Jewry was a bad change for the charming scenery he had left at Zuylestein.

About this epoch was first conceived the idea of an Institution, that afterwards obtained the appellation of the Humane Society; and of which here follows a brief history. Many celebrated writers, both at home and abroad, had been long aware of the fallacy of the received *criteria* of dissolution; and a paper was read, several years ago, before the Royal Society, pointing out the probabilities of a multitude of persons being buried, while *animation was suspended only*, and not destroyed. No proper plan, however, was formed, or even thought of, until Dr. Cogan, in 1773, profiting by what he had seen accomplished with great ease in Holland, conceived the propriety, as well as practicability, of establishing a similar institution in England. He accordingly, in the course of that year, translated and published "Memoirs of the Society instituted at Amsterdam, for the Recovery of Persons apparently Drowned." To this was appended the rules, regulations, and premiums, of this very laudable association.

A few years after this, Dr. Cogan returned to Holland, and soon after undertook a journey into Germany. On this occasion, in company with a friend, he proceeded along the banks of the Rhine, as far as Frankfort. He afterwards descended that noble river, and enjoyed all its luxuriant, romantic, and beautiful prospects. On his coming back to England, he arranged his notes; and, in 1791, published two very entertaining and interesting volumes, in the form of letters.

In 1794, appeared a volume by him in quarto, entitled, "The Works of the late Professor Camper, on the Connexion between the Science of Anatomy, and the Arts of Drawing, Painting, Statuary, &c." This was a translation. In 1800, he published a "Philosophical Treatise on the Passions;" and, in 1807, an "Ethical Treatise on the Passions." In 1812, he appears to have again recurred to the studies of his earlier days; for, in the course of that year, appeared "Disquisitions on the Characteristic Excellencies of Christianity.—To conclude the literary portion of his life, it can be here asserted, from undoubted authority, for the first time, that he was the author of "John Buncle, jun."

It remains now to be mentioned, that Dr. Cogan always entertained a taste for agriculture. To indulge this, he obtained a considerable spot of land at South Wraxall, near Bath, on which he resided for some years. While there, he displayed no common degree of talent in the management of his corn, and grass and cattle; and is said to have farmed to considerable advantage. Indeed, if we are to judge by the premiums obtained by him, he must have excelled most of his neighbours in several branches of rural economy.

Meanwhile, time slipped silently away, and Dr. Cogan, who had lost his wife many years before, had now become a very old man. Yet he appears to have been still exempt from that debility, and those arthritic pains, which generally accompany extreme age. His death occurred at the house of his younger brother, the Rev. E. Cogan, a respectable dissenting minister, who resides at Higham Hill, Walthamstow. The immediate cause of his demise appeared to be a cold, which was accompanied by an asthma, to the latter of which he had been liable, almost every winter, for many years past. The vigour of his mind, however, remained unimpaired to the last. Conscious of his approaching end, he conversed with his usual vivacity, and looked forward to death with a serenity and composure that excited the admiration of all who beheld him. His departure was perfectly easy, for he expired without a struggle or a groan, after having participated in a slight refreshment, in which he seemed to find satisfaction.

Thus died, on February 2, 1818, when he had nearly completed the 82d year of his age, Dr. Thomas Cogan, a man every way respected and estimable. He chiefly dedicated his studies to theology and morals; and his active exertions to the advancement of agriculture. He delighted in travelling into foreign countries, with a view of attaining information for himself, and instruction for others; and it was owing to this laudable species of curiosity that he was enabled to become one of the founders of the Royal Humane Society.

Dr. Cogan had a very agreeable vein for light poetry, and was the author of several humorous songs; among the rest, one with the old burthen—"And a begging we will go," which used to be sung at the anniversaries of the Humane Society.

---

*Adv.*]—Dr. Adams is preparing a new edition of his Memoirs of the late John Hunter, and will be thankful for any particulars he may have omitted, or a correction of any errors which may have escaped. He desires particularly to thank "an Old Hunterian," for his friendly hints, and to assure him they shall be attended to.

---

Observations on a Stridulous Affection of the Bowels, and on some Varieties of Spinal Disease, with an Appendix of Cases, by J. Bradley, M.D. are in the press.

Mr. S. F. Tray, apothecary and teacher of botany and materia medica, has in the press, a Supplement to the several Pharmacopœias, containing the medical uses of all such plants as have been hitherto examined, and an arrangement of their uses; a glossary of the terms and contractions used in prescriptions; formulæ arranged in classes; botanical practice of medicine, offered as hints to regular practitioners to improve the art.

Dr. Speer will shortly publish, in a small volume, "General Views, relating to the Stomach, its Fabric, Functions, &c."

*Adv.*]—Dr. Davis will commence a fresh Course of his Lectures, on the Theory and Practice of Midwifery, and on the Diseases of Women and Children, on Tuesday evening, the 7th of April, at 87, Hatton-garden.

Mr. Salisbury will commence a Course of Botanical Lectures and Excursions in the second week in April.

### REPORT OF DISEASES.

The returning cold has proved dreadful to the phthysical patients who ventured from home during the mild intervals of the preceding month. We wish much that our correspondents would inform us, how far they can confirm our former remark relative to the unusual despondency of those who labour under this, for the most part, flattering complaint. Old people also suffer greatly with asthmas. We have had the rare instance of gout in a work-house pauper. It has been somewhat severe, and was greatly relieved by brisk purgatives: the patient is now convalescent. Our case of Tic Douloureux appears permanently cured, or likely to be easily relieved, on every return, by a very small dose of Extr. Belladonnæ.

Small-pox has rarely appeared, and is almost confined to children. We have not met with a case of scarlatina, or measles; but, hooping-cough seems increasing. Some children have been seized with dysentery in the same houses in which a low fever has prevailed among others of the family. In others, the nettle-rash has been almost endemic.

A delicate female was seized with violent pains in her side, and in the urinary organs, with symptoms of hysteria. All these instantly gave way to the lancet, though hysteria in such a subject would, at one time, have been thought a sufficient objection. A male subject, beyond the age of sixty, and of intemperate habits, has been troubled with hæmoptoe, and violent pains in the head. These symptoms continued at intervals for six weeks. The pain over the forehead increased to an alarming degree, when he was suddenly relieved by Epistaxis. The relief was temporary, and, the pain returning, he submitted to cupping, which proved completely successful. We have dwelt on this case, to show the provision which Nature sometimes makes in favour of subjects who entirely neglect themselves. That truly respectable practitioner, Mr. Peter Matthias, always fearful of plethora, and its consequences, had refused himself wine, and was frequently cupped; yet a very short illness, we believe, in the abdominal cavity, proved fatal.

Most of the diseases continue inflammatory; but, as far as our observation extends, this diathesis is neither so universal nor so severe as in the beginning of the year. That intense pain in the back which is so often the fore-runner of the malignant ulcer of the uterus, the Reporter now always treats with cupping to the loss of a very large quantity of blood, and with success; but, whether any of these cases would really have ended in that melancholy disease cannot be determined.

### METEOROLOGICAL JOURNAL,

By Messrs. WILLIAM HARRIS and Co. 50, Holborn, London.

*From the 20th February to the 19th March, inclusive.*

| Day of Month. | Moon. | Rain gauge | THERM. | | | BAROM. | | De Luc's HYGROM. | | | Wind. | | Atmospheric Variation. | | |
|---|---|---|---|---|---|---|---|---|---|---|---|---|---|---|---|
| | | | | | | | | Dry | Damp | | | | | | |
| Feb. | | | | | | | | | | | | | | | |
| 20 | | | 39 | 47 | 36 | 29·73 | 29·81 | | 23 | 20 | NE | NE | Rain | | |
| 21 | ○ | | 39 | 41 | 34 | 29·63 | 29·45 | | 21 | 17 | W | W | Rain | | |
| 22 | | | 38 | 32 | 31 | 29·39 | 29·35 | | 15 | 15 | S va. | S va. | Clo. | Snow | |
| 23 | | | 37 | 39 | 33 | 29·65 | 29·57 | | 17 | 19 | SW | S | Clo. | Rain | |
| 24 | | | 35 | 42 | 37 | 29·65 | 29·66 | | 27 | 27 | W | SSW | Fine | | |
| 25 | | | 39 | 44 | 35 | 29·59 | 29·50 | | 24 | 20 | W | SSW | Fine | | Rain |
| 26 | | | 37 | 43 | 37 | 29·44 | 29·79 | | 14 | 11 | W | W | Fine | | |
| 27 | ◑ | | 39 | 43 | 39 | 29·47 | 29·48 | | 19 | 16 | SSW | WSW | Rain | | Fine |
| 28 | | | 41 | 44 | 41 | 29·53 | 29·41 | | 15 | 14 | W | SW | Clo. | Fine | Clo. |
| Mar. | | | | | | | | | | | | | | | |
| 1 | | | 44 | 47 | 39 | 29·62 | 29·61 | | 8 | 10 | SW | SSW | Fine | Clo. | |
| 2 | | ,20 | 42 | 47 | 39 | 29·64 | 29·65 | | 12 | 15 | SW | SW | Fine | | Rain |
| 3 | | ,11 | 41 | 52 | 37 | 29·77 | 29·48 | | 17 | 12 | SSW | S va. | Fine | Clo. | |
| 4 | | | 38 | 43 | 43 | 29·39 | 28·83 | | 16 | 13 | SW | SE va. | Fine | | Rain |
| 5 | | ,29 | 44 | 47 | 40 | 28·95 | 29·90 | | 15 | 17 | S | S va. | Fine | Rain | Fine |
| 6 | | ,05 | 42 | 46 | 38 | 29·31 | 29·46 | | 15 | 17 | SW | SSW | Fine | Show-ery | Fine |
| 7 | ● | ,09 | 42 | 45 | 40 | 29·27 | 28·89 | | 19 | 17 | SSW | SW | Rain | | |
| 8 | | | 43 | 45 | 35 | 28·98 | 29·31 | | 14 | 12 | WNW | W va. | Fine | Clo. | Fine |
| 9 | | | 37 | 41 | 33 | 29·41 | 29·37 | | 12 | 6 | SW | WSW | Fine | | Rain & Snow |
| 10 | | ,20 | 35 | 40 | 35 | 29·36 | 29·37 | | 16 | 12 | SW | W | Fine | Snow | Fine |
| 11 | | ,09 | 37 | 44 | 34 | 29·48 | 29·25 | | 11 | 12 | W va. | SSE | Fine | Clo. | Rain & Snow |
| 12 | | | 35 | 42 | 36 | 28·92 | 29·11 | | 16 | 12 | W | SW | Fine | Clo. | Fine |
| 13 | | | 37 | 45 | 35 | 29·32 | 29·77 | | 18 | 12 | N | W | Fine | Show-ery | Rain |
| 14 | | ,05 | 36 | 40 | 40 | 29·86 | 29·80 | | 15 | 17 | W | SSW | Fine | Rain | Fine |
| 15 | ◐ | ,06 | 42 | 46 | 39 | 29·43 | 29·39 | | 15 | 18 | SSE | W | Fine | Rain | Clo. |
| 16 | | .07 | 40 | 46 | 42 | 29·40 | 29·71 | | 18 | 11 | W | SW | Clo. | Hail | Clo. |
| 17 | | | 45 | 48 | 41 | 29·74 | 30·04 | | 17 | 11 | WNW | SW | Show-ery | Clo. | Fine |
| 18 | | .06 | 44 | 51 | 45 | 30 17 | 30·02 | | 16 | 11 | SSW | SW | Clo. | Fine | |
| 19 | | ,03 | 47 | 53 | 47 | 29·98 | 29·80 | | 15 | 17 | SW | SW | Clo. | | Fine |

